# Effects of aerobic exercise on integrated cardiovascular health and energy metabolism in patients with type 2 diabetes mellitus: study protocol for a randomized controlled trial

**DOI:** 10.3389/fendo.2026.1748335

**Published:** 2026-01-21

**Authors:** Shihe Wang, Zhouyu Liu, Ning Feng, Yaodong Guo, Qi Chen, Yiyang Cao, Xuelu Li, Xiao Liu, Zhiwei Yan

**Affiliations:** 1 Liaoning Provincial Sports Development Center, Shenyang, Liaoning, China; 2 The First Affiliated Hospital of Dalian Medical University, Dalian, Liaoning, China; 3 College of Exercise and Health, Shenyang Sport University, Shenyang, Liaoning, China; 4 Provincial University Key Laboratory of Sport and Health Science, School of Physical Education and Sport Sciences, Fujian Normal University, Fuzhou, Fujian, China; 5 The QUEEN MARY School, Jiangxi Medical College, Nanchang University, Nanchang, Jiangxi, China; 6 Department of Breast Surgery and Oncology, The Second Hospital of Dalian Medical University, Dalian, Liaoning, China; 7 Department of Cardiology, Sun Yat‐sen Memorial Hospital of Sun Yat‐sen University, Guangzhou, Guangdong, China; 8 Cardiovascular and Metabolic Disorders Program, Duke-National University of Singapore Medical School, Singapore, Singapore

**Keywords:** aerobic exercise, cardiovascular health, energy metabolism, integrative physiological function, T2DM

## Abstract

**Objective:**

Type 2 diabetes mellitus (T2DM) induces integrated cardiovascular and metabolic impairments. The comprehensive effect of aerobic exercise on these systemic alterations remains unclear. Therefore, we designed a randomized controlled trial to investigate its impact on integrated cardiovascular health — specifically assessed by nitroglycerin-mediated dilation (NMD) and myocardial global work efficiency (GWE) — and systemic energy metabolism in patients with T2DM.

**Methods:**

This study is a randomized, single-assessor-blind, parallel-group, two-arm controlled trial that will enroll 74 patients with T2DM. Participants will be randomly assigned in a 1:1 ratio to either a 12-week supervised aerobic exercise group (55–75% of HR_peak_, three times per week) or a non-exercise control group. The primary outcomes are the changes from baseline to 12 weeks in NMD and GWE. Secondary outcomes include key cardiometabolic indicators (energy metabolism, insulin levels), office and 24-hour ambulatory blood pressure, cardiovascular risk factors, 24-hour movement behavior, cardiopulmonary function, arterial stiffness (baPWV), vascular endothelial function (FMD), cardiac electrical activity, echocardiographic parameters, and self-reported fatigue. Additional exploratory variables include markers of myocardial injury and skeletal muscle injury, circulating markers of oxidative stress and inflammation, central fatigue biomarkers, and levels of cardiovascular-related hormones. Recruitment began in June 2025 and is currently ongoing.

**Discussion:**

This study provides an integrated physiological perspective on how 12-week moderate-intensity aerobic exercise influences cardiovascular function and systemic metabolism in patients with T2DM. We hypothesize that 12 weeks of aerobic exercise will significantly improve NMD and GWE, concurrent with enhanced metabolic flexibility and reduced fatigue compared to the non-exercise control group.

## 1 Introduction

Type 2 diabetes mellitus (T2DM) is a metabolic disorder characterized by insulin resistance, which leads to impaired glucose metabolism and a subsequent loss of metabolic flexibility (1). Cardiovascular disease is the leading cause of morbidity and mortality among patients with T2DM (2–4). Through mechanisms including metabolic dysregulation, oxidative stress, and inflammation, T2DM induces pathological remodeling and functional impairment of the cardiovascular system (5). Patients with T2DM frequently present with significant diastolic dysfunction and autonomic imbalance, accompanied by myocardial hypertrophy and fibrosis, which are hallmarks of adverse myocardial remodeling (6). Furthermore, T2DM can induce myocardial metabolic inflexibility, leading to dysregulated energy metabolism that accelerates cardiac dysfunction (7). In this context, myocardial Global Work Efficiency (GWE) serves as a sensitive and early indicator for assessing myocardial energetics (8). Vasculopathy is another common complication of T2DM. Patients frequently exhibit increased arterial stiffness, pathological vascular remodeling, and impaired endothelium-dependent vasodilation (9). Notably, as research has confirmed the prevalence of vascular smooth muscle dysfunction in T2DM, Nitroglycerin-Mediated Dilation (NMD) is employed to specifically evaluate vascular smooth muscle function. These changes contribute to the dysregulation of peripheral resistance and an elevated risk of atherosclerosis (10). Consequently, the pathological crosstalk between cardiac and vascular abnormalities in T2DM leads to a significant elevation in systolic blood pressure (SBP), ultimately disrupting blood pressure homeostasis (11). This underscores the systemic damage that T2DM imposes on the cardiovascular system as a whole. Furthermore, accumulating evidence indicates that patients with T2DM commonly exhibit reduced peak oxygen uptake (VO_2peak_). As an integrative measure of the coordinated function of the cardiovascular, respiratory, and musculoskeletal systems, VO_2peak_ is the strongest predictor of integrated cardiovascular function in this population (12, 13). From the perspective of integrative physiology, dysfunction in a single organ system inevitably compromises overall systemic function. This suggests that T2DM induces pathological inter-organ interactions via shared mechanisms, resulting in global cardiovascular dysfunction rather than isolated organ damage. Therefore, the focus of therapeutic interventions for T2DM should expand from targeting single organs to enhancing integrated physiological function to achieve more comprehensive clinical benefits.

Aerobic exercise (AE) is a well-established non-pharmacological intervention for improving cardiovascular health and ameliorating metabolic abnormalities, and it is clinically recommended as a cornerstone of T2DM management. Specifically, AE has been shown to enhance insulin sensitivity and improve glucose metabolism (14). In addition, AE can attenuate T2DM-induced cardiovascular remodeling and functional impairments (15). However, existing studies have primarily focused on the effects of AE on individual cardiovascular organs or isolated metabolic pathways. There is limited evidence regarding its impact on integrated cardiovascular function and systemic metabolism. Thus, there is a compelling need to investigate the role of AE in promoting integrative physiological function in this population.

Although regular AE can improve metabolic and cardiovascular health in patients with T2DM, it is noteworthy that this population generally has insufficient levels of physical activity (PA) (16). Low PA can trigger a cascade of adverse physiological responses, including impaired energy metabolism, decreased insulin sensitivity, reduced exercise tolerance, and increased cardiovascular disease risk, thereby perpetuating or exacerbating the pathological progression of T2DM (17). The reasons for insufficient PA among individuals with T2DM are multifactorial, with fatigue being recognized as a key contributor. The pathogenesis of fatigue is complex, involving disturbances in energy metabolism, oxidative stress, and inflammation (18). Specifically, fatigue in this population manifests through both central and peripheral pathways: centrally, it involves disturbances in neurotransmitter balance, while peripherally, it is characterized by skeletal muscle injury and oxidative stress. While regular AE has been shown to alleviate fatigue in patients with T2DM, the underlying mechanisms remain unclear, and most studies have lacked objective biomarkers to quantify fatigue.

We hypothesize that a 12-week moderate-intensity aerobic exercise intervention will significantly enhance integrated cardiovascular function, specifically by improving GWE and NMD, while concurrently restoring systemic energy metabolism and metabolic flexibility in patients with T2DM. Furthermore, regarding the mechanisms of fatigue alleviation, we hypothesize that aerobic exercise will mitigate fatigue via a dual pathway: centrally, by modulating the balance of neurotransmitters (including serotonin, dopamine, and GABA), and peripherally, by reducing skeletal muscle injury biomarkers (CK and Myoglobin) and oxidative stress.

## 2 Materials and methods

### 2.1 Research design

This study is a single-assessor-blind, parallel-group, two-arm randomized controlled trial. Participants will be randomly assigned in a 1:1 ratio to either a 12-week aerobic exercise group (AE group) or a non-exercise control group (Con group). Clinical assessments will be performed at baseline (pre-intervention) and at the 12-week follow-up. A detailed schedule of enrollment, interventions, and assessments is presented in **Table 1**. Considering the minimum clinically effective dose of exercise, adherence to the intervention will be defined as the completion of at least 80% of the prescribed exercise sessions. Participants who do not meet this threshold will be categorized as non-adherent. To minimize attrition bias, all participants, regardless of their adherence or completion of the intervention, will be followed up and included in the primary analysis according to the intention-to-treat (ITT) principle. Written informed consent will be obtained from all participants before enrollment. The reporting of this trial will adhere to the Consolidated Standards of Reporting Trials (CONSORT) statement.

**Table 1:** Participant timeline: schedule of enrollment, interventions, and assessments.

Trial period	Enrollment		Post-randomization			Close-out
Timepoint	Screening *(Day-12 to 0)*	Baseline *(Day0)*	Intervention Period *(Weeks 1-6)*	Intervention Period *(Day42)*	Intervention Period *(Weeks 6-12)*	*Week 12 (± 3 days)*
Enrollment:						
Eligibility screen	X					
Informed consent	X					
Medical history	X					
Randomization		X				
Intervention:						
Aerobic Exercise Group		X	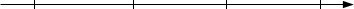			X
Control Group		X	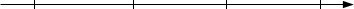			X
Assessments:						
Primary outcomes						
nitroglycerin-mediated dilation		X				X
myocardial global work efficiency		X				X
Secondary outcomes						
Energy Metabolism		X				X
Peak Heart Rate (HR_peak_)		X		X		X
Traditional Cardiovascular Risk Factors		X				X
Cardiovascular & Cardiopulmonary Function		X				X
Self-reported fatigue assessment		X				X
24-hour physical activity monitoring		X				X
Circulating biomarkers		X				X
Safety monitoring						
Adverse Events		X	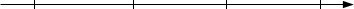			X

### 2.2 Research setting

**Figure 1:**
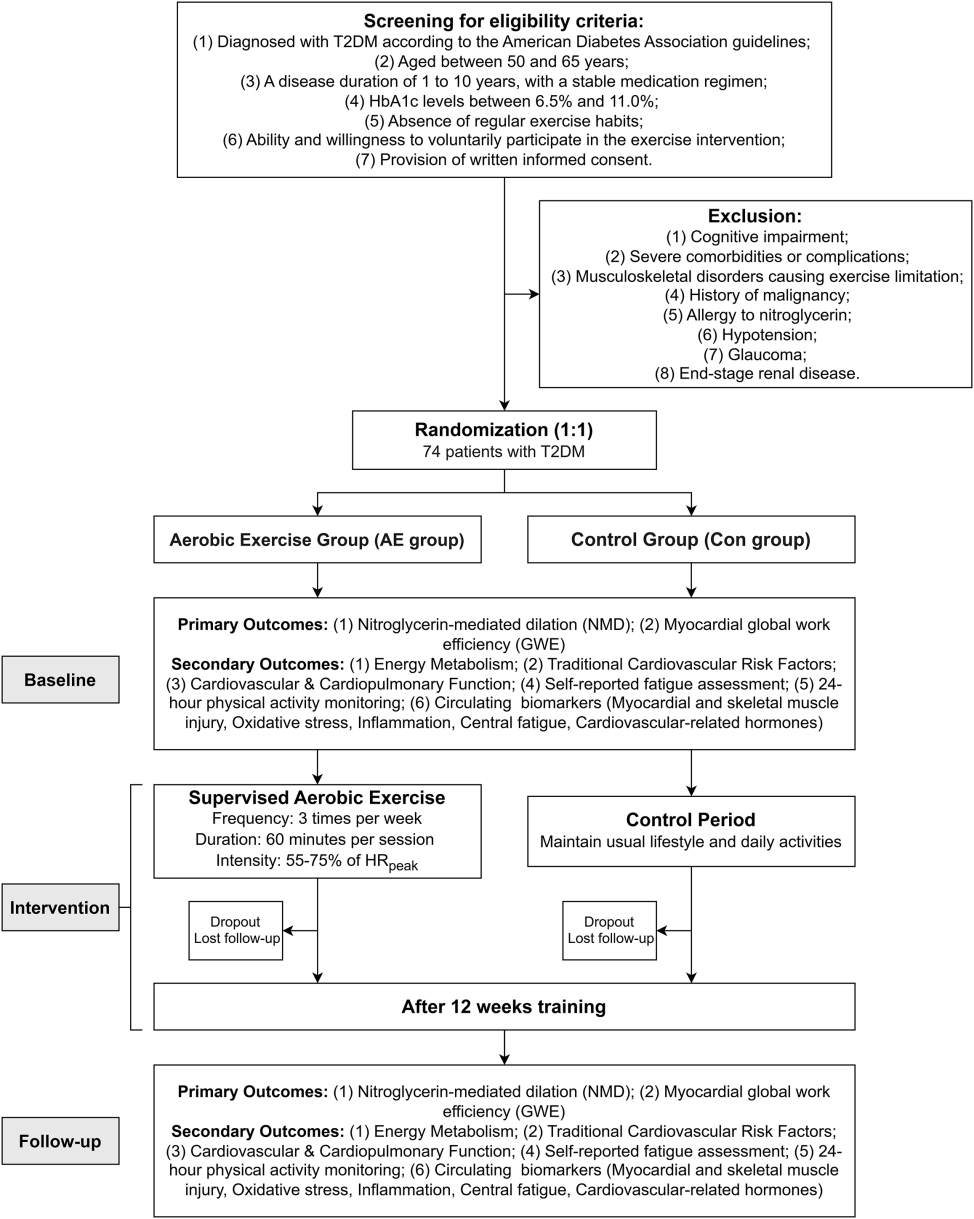
Flowchart of study procedures.

A total of 74 participants with T2DM will be recruited from local communities in Shenyang, China in collaboration with community physicians. Recruitment strategies will include the distribution of posters, flyers, brochures, and health education lectures. Clinical physicians will confirm the diagnosis and assess eligibility for all potential participants. The exercise intervention will be conducted at a fitness and rehabilitation center, while all clinical assessments will take place at a partner hospital and associated laboratories. Baseline and follow-up assessments will be conducted by trained research staff who are blinded to group allocation, and the exercise intervention will be supervised by certified clinical exercise physiologists. Participant recruitment commenced in June 2025. The study execution period is from June 30, 2025, and is currently ongoing (**Figure 1**).

### 2.3 Inclusion criteria

Participants will be eligible for inclusion if they meet the following criteria: (1) diagnosed with T2DM according to the American Diabetes Association (ADA) guidelines (19); (2) aged between 50 and 65 years; (3) a disease duration of 1 to 10 years, with a stable medication regimen (including glucose-lowering agents, antihypertensives, and statins) for at least 3 months prior to enrollment; (4) HbA1c levels between 6.5% and 11.0%; (5) absence of regular exercise habits, defined as performing less than 150 minutes of moderate-intensity physical activity per week and being categorized as having a “Low” physical activity level according to the International Physical Activity Questionnaire (IPAQ) scoring protocol; (6) ability and willingness to voluntarily participate in the exercise intervention; and (7) provision of written informed consent.

### 2.4 Exclusion criteria

Participants will be excluded from the study if they meet any of the following criteria: (1) cognitive impairment; (2) severe comorbidities or complications such as heart failure, autonomic neuropathy, or stroke; (3) musculoskeletal disorders causing exercise limitation; (4) history of malignancy; (5) allergy to nitroglycerin; (6) hypotension; (7) glaucoma; or (8) end-stage renal disease.

### 2.5 Sample size calculation

The sample size calculation is based on the two co-primary outcomes: NMD and GWE. The calculation is for a superiority trial, designed to achieve 80% statistical power at a two-sided significance level (α) of 0.05. No adjustment for multiplicity was applied to the co-primary outcomes (NMD and GWE) because they represent distinct physiological domains and address separate, independent research questions. NMD assesses the structural integrity and function of peripheral vascular smooth muscle (endothelium-independent pathway), whereas GWE serves as a specific metric for central myocardial energetics and metabolic efficiency. In the pathophysiology of T2DM, peripheral vascular remodeling and myocardial metabolic inflexibility are distinct complications that may progress asynchronously and respond to intervention via different mechanisms. Therefore, to capture the distinct effects of aerobic exercise on these independent physiological systems, each outcome was evaluated at a two-sided significance level of 0.05. All calculations were performed using PASS (Power Analysis and Sample Size) software. For GWE, as no direct data on the effects of AE exist, parameters are derived from a study on the effects of GLP-1 receptor agonists (GLP-1 RAs) in patients with T2DM. We hypothesized that the effect of AE would be 75% of that observed with GLP-1 RA treatment (20). This yields an estimated mean between-group difference (δ) of 2.7% with a projected standard deviation (σ) of 4.0%. This corresponds to a standardized effect size (Cohen’s d) of approximately 0.68, representing a “moderate-to-large” effect size. This estimation is considered conservative given that lifestyle and exercise interventions have previously demonstrated comparable or superior efficacy to medical interventions in key physiological domains. For example, the Diabetes Prevention Program showed that lifestyle intervention was more effective than metformin for the metabolic profile (21).

Based on these parameters, a minimum of 34 participants per group (n=68 total) is required to detect a significant difference. For NMD, the estimation is based on data from Ziegler et al., we estimated a mean intervention effect (δ) of 4.0% with a standard deviation (σ) of 5.6% (22). This calculation requires a minimum of 32 participants per group (n=64 total). To adequately power the study for both co-primary outcomes, we will use the larger of the two estimates. Therefore, the sample size is driven by the requirements for NMD. To account for a potential 10% dropout rate, we will recruit 37 participants per group, bringing the total sample size to 74.

### 2.6 Random assignment and blinding

Following baseline assessments, participants will be randomly assigned in a 1:1 ratio to either the AE group or the Con group. The randomization sequence will be generated using a computer-based random number generator (Microsoft Excel) by an independent statistician who is not involved in participant recruitment or data collection, employing a simple randomization method. Allocation concealment will be maintained using sequentially numbered, opaque, sealed envelopes. These envelopes will be kept by a designated research assistant and will be opened only after baseline assessments are completed, thereby minimizing selection bias.

This study employs a single-assessor-blind design. To maintain the blind, a rigorous single-blinded assessor design will be implemented where all participants are assigned unique alphanumeric identification codes for data collection and analysis. Specifically, imaging specialists (echocardiographers and vascular technicians) will perform scans and post-processing analyses using only these codes, and will be strictly trained to refrain from discussing intervention allocation or personal perceptions of improvement with participants during examinations to prevent inadvertent unblinding. Similarly, biochemical analysts will process blood samples labeled solely with identification codes, ensuring they remain unaware of group allocation or sampling time points. Furthermore, statistical analysis will be conducted by an independent statistician using de-identified datasets with dummy group labels. Access to unblinded data will be restricted exclusively to the clinical exercise physiologists delivering the intervention and the Data Safety Monitoring Board responsible for monitoring adverse events. This design ensures methodological rigor, minimizes bias, and enhances the reliability and validity of the study findings.

### 2.7 Intervention

The exercise intervention for the AE group is structured according to the FITT-VP principle (Frequency, Intensity, Time, Type, Volume, Progression). The protocol aligns with the American College of Sports Medicine (ACSM) guidelines for exercise in patients with T2DM (23). All training sessions will be supervised by certified clinical exercise physiologists. Considering the significant effects of AE on glucose homeostasis, all supervised sessions will be scheduled 1–2 hours postprandially to prevent exercise-induced hypoglycemia.

The exercise intervention consists of a 12-week supervised program with three sessions per week. Each session comprises a 5-minute warm-up (50%–60% of HR_peak_), 50 minutes of aerobic exercise (55%–75% of HR_peak_), and a 5-minute cool-down (50%–60% of HR_peak_). Given the progressive nature of cardiorespiratory adaptations to exercise training, the 12-week program will be divided into an initial phase (Weeks 1–6) and a secondary phase (Weeks 7–12). Cardiopulmonary exercise testing (CPET) will be performed at baseline and at the end of Week 6 to assess cardiorespiratory fitness and to prescribe exercise intensity based on the heart rate peak (HR_peak_). The HR_peak_ is identified as the heart rate corresponding to the achievement of VO_2peak_ during the CPET. For participants receiving heart rate–modulating medications (e.g., β-blockers or non-dihydropyridine calcium channel blockers) or those exhibiting chronotropic incompetence with a blunted heart rate response, exercise intensity will be prescribed using a combination of the power output (Watts) derived from the CPET and the Borg Rating of Perceived Exertion (RPE; target range 12–14). This approach ensures the delivery of a consistent and relatively moderate-intensity training stimulus. Throughout the intervention, participants in the AE group will wear heart rate monitors Polar H7 (Polar Electro, Kempele, Finland) to facilitate real-time monitoring and dynamic adjustment of exercise intensity.

Participants assigned to the control group (Con group) will not receive any supervised or structured exercise intervention. To ensure a clear distinction from the AE group, they will be explicitly instructed to maintain their habitual lifestyle patterns throughout the 12-week study period. To objectively monitor adherence to these instructions, physical activity levels will be assessed weekly using the International Physical Activity Questionnaire (IPAQ). Any deviations from baseline activity patterns will be documented.

Throughout the 12-week intervention, all participants will be required to maintain their baseline medication regimens. Dosage adjustments will be permitted only when medically necessary for safety reasons (e.g., in cases of severe hypoglycemia or uncontrolled blood pressure), and any such modifications will be meticulously documented.

### 2.8 Measurement

Comprehensive clinical assessments—including metabolic, cardiovascular, cardiopulmonary, fatigue, and skeletal muscle tests—will be performed at baseline and at the 12-week follow-up. With the exception of the oral glucose tolerance test and associated 2-hour postprandial glucose measurement, all assessments will be conducted in the morning after a minimum 12-hour overnight fast. Participants will be instructed to abstain from alcohol, caffeine for 24 hours and withhold their morning medications on the day of assessment to minimize pharmacological interference. To mitigate the confounding effects of acute exercise, post-intervention assessments will be scheduled 48 hours after the final training session. For biochemical analyses, venous blood samples (approximately 6 mL) will be drawn from an antecubital vein into serum separation tubes. The tubes will be gently inverted and then allowed to clot at room temperature for 30–60 minutes. The samples will then be centrifuged at 3000 rpm for 10 minutes at 4°C, and the resulting serum will be aliquoted and stored at –80°C until analysis. To minimize inter-assessor and instrument variability, all measurements will be performed by the same trained technician using the same equipment at both time points.

### 2.9 Safety considerations and adverse event monitoring

Safety protocols will be rigorously enforced throughout the trial in accordance with the latest Consensus Statement from the American College of Sports Medicine (ACSM) (23). Prior to each training session or CPET assessment, participants will undergo a mandatory health screening, including an assessment of subjective well-being (e.g., dizziness, palpitations, excessive fatigue). Capillary blood glucose measurement will be performed conditionally, specifically when participants report symptoms suggestive of acute glycemic dysregulation (e.g., lightheadedness, shakiness, or diaphoresis) or general malaise. Sessions will be postponed if a participant reports significant subjective discomfort, or if measured blood glucose levels are<3.9 mmol/L, or >16.7 mmol/L, or >13.9 mmol/L with concurrent ketonuria or symptoms of metabolic decompensation. In the event of hypoglycemia, participants will ingest fast-acting carbohydrates and undergo re-testing after 15 minutes; the session will proceed only once glucose levels have returned to a safe range. Criteria for the immediate termination of a CPET or training session will follow ACSM guidelines, including the onset of angina-like symptoms, a fall in systolic blood pressure of more than 10 mmHg despite increasing workload, an excessive hypertensive response (systolic blood pressure >250 mmHg or diastolic blood pressure >115 mmHg), severe shortness of breath, dizziness, signs of poor perfusion, clinically significant arrhythmias, or marked ST-segment abnormalities. All adverse events, including musculoskeletal injuries, cardiovascular symptoms, and any unanticipated reactions, will be documented in standardized case-report forms by the supervising clinical exercise physiologist immediately upon occurrence. An independent Data Safety Monitoring Board (DSMB), not involved in the delivery of the intervention, will adjudicate all events to evaluate severity, relatedness, and the need for protocol modification or participant withdrawal.

### 2.10 Primary outcomes

#### 2.10.1 Vascular smooth muscle function

Vascular smooth muscle function will be assessed using the same ultrasound device (unexp18g, UNEX, Japan) following a procedure similar to that of the FMD test. After at least 15 minutes of rest, participants will receive 75 µg of sublingual nitroglycerin, and the maximal dilation of the same brachial artery segment will be measured. NMD will be calculated using the formula:


$$\text{NMD\%}=\frac{\text{maximal post-nitroglycerin diameter }-\text{ baseline arterial diameter}}{\text{baseline arterial diameter}}\times 100\%.$$

#### 2.10.2 Speckle-tracking and myocardial work analysis

Two-dimensional echocardiographic images of the left ventricle—including short-axis views at the basal, mid, and apical levels, as well as apical four-chamber, two-chamber, and three-chamber views—will be analyzed using EchoPAC software (Version 203). Post-processing will be performed to obtain left ventricular global longitudinal strain (LS), circumferential strain (CS), radial strain (RS), and rotational/torsional angles. Brachial artery pressure values recorded before imaging will be entered into the software, which will automatically generate left ventricular pressure–strain loops and calculate myocardial work parameters, including global work index (GWI), global constructive work (GCW), global wasted work (GWW), and global work efficiency (GWE).

### 2.11 Secondary outcome

#### 2.11.1 Glucose metabolism

The following glucose metabolism parameters will be measured using a fully automated biochemical analyzer: fasting blood glucose, postprandial 2-hour blood glucose, and glycated hemoglobin (HbA1c). Blood collection and storage will be performed as previously described. Fasting glucose and postprandial glucose will be measured using corresponding assay kits, while HbA1c will be quantified via high-performance liquid chromatography (HPLC).

#### 2.11.2 Insulin levels

Fasting insulin levels will be measured using a commercial enzyme-linked immunosorbent assay (ELISA) kit, following the manufacturer’s protocol. The homeostasis model assessment of insulin resistance (HOMA-IR) index will be calculated from fasting glucose and insulin values using the standard formula:


HOMA-IR=Fasting blood glucose (mmol/L)×Fasting insulin (µU/mL)/22.5

#### 2.11.3 Oral glucose tolerance test

Venous fasting blood samples will be collected the following morning to avoid prolonged fasting that could lead to reactive hyperglycemia and interfere with diagnostic accuracy. A glucose solution will be prepared by dissolving 75 g of anhydrous glucose powder in 150 mL of water, and participants will be instructed to consume the entire 150 mL solution within 5 minutes. The start of ingestion will be recorded as time zero. Venous blood samples will then be collected at fasting, 60 and 120 minutes after ingestion to assess blood glucose changes.

#### 2.11.4 Lipid metabolism

Lipid metabolism parameters, including high-density lipoprotein cholesterol (HDL-C), low-density lipoprotein cholesterol (LDL-C), triglycerides (TG), and total cholesterol (TC), will be measured using a fully automated biochemical analyzer. Blood collection and storage will follow the standard protocol described above, and lipid profiles will be assessed using corresponding assay kits.

#### 2.11.5 Energy metabolism

One hour after consuming a standardized meal, participants will undergo a 30-minute resting respiratory exchange ratio (RER) test using a metabolic gas analyzer (Cortex Meta Max 3B, CORTEX, Germany) while lying supine. The mean RER will be calculated after excluding data from the first and last 5 minutes. Subsequently, participants will perform 30 minutes of AE at 60% of their individual VO_2_peak to measure exercise RER. During the test, workload will be adjusted based on oxygen consumption to ensure the target intensity is maintained. Metabolic flexibility will be evaluated using the difference between exercise and resting RER ( ΔRER=RER_exercise−RER_rest), with a larger ΔRER indicating greater metabolic flexibility.

#### 2.11.6 Cardiovascular risk factors

Height, body weight, waist circumference, and hip circumference will be measured using a stadiometer, digital scale, and non-elastic measuring tape to assess cardiovascular risk factors. Body mass index will be calculated as $\;\text{body weight (kg)}/\text{height squared (}{\text{m}}^{2})$. Waist-to-hip ratio (WHR) will be calculated as  waist circumference (cm)/hip circumference (cm). Subcutaneous adipose tissue thickness will be measured at three sites—triceps, subscapular, and abdominal regions—using a skinfold caliper. In addition, an ultrasound body composition analyzer (BodyMetrix BX-2000, Intelametrix, Livermore, USA) will be used to assess fat thickness at seven anatomical sites, including the triceps, subscapular, mid-axillary, abdominal, pectoral, suprailiac, and thigh regions.

#### 2.11.7 Echocardiography

After resting in the supine position for 5 minutes, participants’ right brachial artery blood pressure will be measured three times and recorded. Participants will then be placed in the left lateral decubitus position, connected to an ECG, and examined using a GE Vivid E95 color Doppler ultrasound system equipped with an M5S transducer. Dynamic images from three consecutive cardiac cycles will be acquired and stored from the following standard echocardiographic views: parasternal long-axis view of the left ventricle, parasternal short-axis views at the basal, mid-papillary, and apical levels, and apical four-chamber, two-chamber, and three-chamber views.

Cardiac Structural and Systolic Function Parameters: From the parasternal long-axis view, left ventricular end-diastolic diameter (LVEDD), end-systolic diameter (LVESD), interventricular septal thickness (IVST), and left ventricular posterior wall thickness (LVPWT) will be measured. Left ventricular remodeling indices—including relative wall thickness (RWT) and left ventricular mass index (LVMI)—will be calculated. Systolic function parameters, including left ventricular ejection fraction (LVEF), stroke volume (SV), and cardiac output (CO), will be obtained using the biplane Simpson’s method from the apical four-chamber and two-chamber views.

Diastolic Function Parameters: Pulsed-wave Doppler (PW) will be used at the mitral valve inflow to measure early (E) and late (A) diastolic filling velocities. Tissue Doppler imaging (TDI) will be applied at the septal and lateral mitral annulus to measure early diastolic myocardial velocity (e’), from which the mean e’ and mean E/e’ ratio will be calculated to evaluate left ventricular diastolic function.

#### 2.11.8 Electrocardiography

A standard 12-lead resting electrocardiogram (ECG) will be recorded using a digital ECG system (EDAN SE-1010, China) after the participant has rested in a supine position for at least 10 minutes. All intervals will be measured from lead II, except for the QT interval, which will be measured in either lead II or V5 (whichever is clearer). The following parameters will be analyzed: P-wave duration (ms), PR interval (ms), QRS duration (ms), and QT interval (ms). The QT interval will be corrected for heart rate (QTc) using Bazett’s formula:


$$\text{QTc}=\text{QT}/\sqrt{\text{RR}}.$$

#### 2.11.9 24-hour ambulatory electrocardiogram

The 24-hour ambulatory electrocardiogram (Holter) will be used to assess cardiac autonomic nervous function. Time-domain indices will include SDNN, RMSSD, and PNN50. Frequency-domain indices will include total power (TP), high-frequency power (HF), low-frequency power (LF), very low-frequency power (VLF), and the LF/HF ratio. Nonlinear indices such as SD1, SD2, and SDRR will also be analyzed to provide a comprehensive evaluation of autonomic modulation.

#### 2.11.10 Arterial stiffness

Arterial stiffness and elasticity will be assessed using an arteriosclerosis detector (BP-203RPEIII, Omron, Japan) to measure brachial–ankle pulse wave velocity (baPWV) and the ankle–brachial index (ABI).

#### 2.11.11 Vascular endothelial function

Flow-mediated dilation (FMD) will be evaluated using an ultrasound vascular imaging system (unexp18g, UNEX, Japan). FMD will be calculated using the formula:


$$\text{FMD\%}=\frac{\text{peak post-hyperemia diameter }-\text{ baseline arterial diameter}}{\text{baseline arterial diameter}}\times 100\%.$$

#### 2.11.12 Blood pressure

Resting office systolic blood pressure (SBP), diastolic blood pressure (DBP), pulse pressure (PP), and rate–pressure product (RPP) will be measured using an automated sphygmomanometer (Omron HEM-3, Omron, Japan). Participants will be instructed to remain seated and relaxed during the measurement. Blood pressure will be measured three times, and the mean value of the last two readings will be used for analysis.

#### 2.11.13 24-hour ambulatory blood pressure

Dynamic blood pressure will be recorded using a noninvasive ambulatory blood pressure monitor (D2 Watch, Huawei, China). Daytime is defined as 06:00–22:00 and nighttime as 22:00–06:00. At least 90% of readings must be valid to ensure data reliability. The monitoring parameters include 24-hour mean SBP and DBP, daytime mean SBP and DBP, and nighttime mean SBP and DBP.

#### 2.11.14 24-hour movement behavior

24-hour movement behavior will be objectively monitored using a triaxial accelerometer (ActiGraph GT3X, USA) worn on the non-dominant wrist. The sampling frequency of the accelerometer will be set to 30 Hz with a recording epoch of 10 seconds. Participants will be instructed to maintain their usual daily routines (e.g., studying, household activities, and leisure activities) and wear the device continuously throughout the monitoring period.

#### 2.11.15 Cardiopulmonary exercise testing

A graded incremental exercise test will be performed to measure participants’ peak oxygen uptake (VO_2peak_). Gas exchange will be analyzed using an indirect calorimeter (Cortex Meta Max 3B, CORTEX, Germany), and exercise load will be applied via a cycle ergometer (Monark 839E, Varberg, Sweden). Participants will perform a 3-minute warm-up at 50 W for men and 35 W for women. Thereafter, the workload will increase by 15 W per minute until the participant reaches volitional exhaustion. Gas analysis data will be collected every 10 seconds, and VO_2peak_ will be determined as the average oxygen uptake during the highest 30-second interval. Throughout the test, both subjective and objective fatigue levels will be monitored using the heart rate monitor Polar H7 (Polar Electro, Kempele, Finland) and the Borg Rating of Perceived Exertion (RPE) scale. Strict safety monitoring will be maintained throughout the test, and the specific criteria for immediate test termination are identical to those detailed in the ‘Safety considerations and adverse event monitoring’ section, adhering to ACSM guidelines. Achievement of VO_2peak_ will be confirmed when two or more of the following criteria are met: (1) a plateau in oxygen consumption despite increasing workload; (2) heart rate within ±10 beats per minute of the age-predicted maximum during the final stage; (3) respiratory exchange ratio (RER) > 1.1; and (4) RPE score ≥ 17.

#### 2.11.16 Myocardial injury biomarkers

The serum levels of cardiac troponin I (cTnI), N-terminal pro-B-type natriuretic peptide (NT-proBNP), and creatine kinase-MB (CK-MB) will be determined using ELISA kits from Shenzhen Edan Instruments Co., Ltd. (m16). The specific experimental procedures will be carried out in strict accordance with the instructions provided with the kits.

#### 2.11.17 Skeletal muscle injury biomarkers

The serum levels of creatine kinase (CK) and myoglobin (Myo) will be measured using ELISA kits from Shenzhen Edan Instruments Co., Ltd. (m16). The specific experimental procedures will be carried out in strict accordance with the instructions provided with the kits.

#### 2.11.18 Circulating vascular function biomarkers

The serum levels of angiotensin-(1–7) [Ang-(1–7)], angiotensin II (Ang II), and endothelin-1 (ET-1) will be determined using ELISA kits from Jiangsu Enzyme Exemption Industry Co., Ltd. (MM-1719H2, MM-0004H2, and MM-0998H1, respectively). Serum nitric oxide (NO) levels will be measured by the microplate method using a kit from Jiangsu Edison Biotechnology Co., Ltd. (ADS-W-N005-96). The specific experimental procedures will be carried out in strict accordance with the instructions provided with the kits.

#### 2.11.19 Circulating oxidative stress biomarkers

Serum reactive oxygen species (ROS) levels will be quantified using a green fluorescence method with a kit from Shanghai Beibo Biotechnology Co., Ltd. (Product No. BB-475015). Serum superoxide dismutase (SOD) and malondialdehyde (MDA) levels will be determined by the microplate method using kits from Jiangsu Edison Biotechnology Co., Ltd. (ADS-W-KY011 and ADS-W-YH002, respectively). Serum levels of nuclear factor erythroid 2-related factor 2 (Nrf2), Kelch-like ECH-associated protein 1 (KEAP1), heme oxygenase-1 (HO-1), glutathione peroxidase 4 (GPX4), and NAD(P)H quinone dehydrogenase 1 (NQO1) will be measured by ELISA using kits from Jiangsu Enzyme Exemption Industry Co., Ltd. (MM-2402H1, MM-50943H1, MM-1508H1, MM-60328H1, and MM-50945H1, respectively). The specific experimental procedures will be carried out in strict accordance with the instructions provided with the kits.

#### 2.11.20 Circulating inflammatory cytokines

Serum concentrations of interleukin-18 (IL-18), interleukin-1β (IL-1β), interleukin-6 (IL-6), tumor necrosis factor-alpha (TNF-α), C-reactive protein (CRP), and NLR family pyrin domain containing 3 (NLRP3) will be determined using ELISA kits from Jiangsu Enzyme Exemption Immunosorbent Assay Industrial Co., Ltd. (MM-0139H1, MM-0181H1, MM-0049H1, MM-0122H1, MM-0135H1, and MM-51822H1, respectively). The specific experimental procedures will be carried out in strict accordance with the instructions provided with the kits.

#### 2.11.21 Central fatigue biomarkers

Serum levels of 5-hydroxytryptamine (5-HT), dopamine (DA), and gamma-aminobutyric acid (GABA) will be measured by ELISA using kits from Nanjing SenBeijia Biological Technology Co., Ltd. (SBJ-H0191-96T, SBJ-H1796-96T, and SBJ-H1124-96T, respectively). The specific experimental procedures will be carried out in strict accordance with the instructions provided with the kits.

#### 2.11.22 Catecholamines

Serum levels of noradrenaline (NA) and adrenaline (A) will be quantified using ELISA kits from Nanjing SenBeijia Biological Technology Co., Ltd. (SBJ-H0141-96T and SBJ-H0965-96T, respectively). The specific experimental procedures will be carried out in strict accordance with the instructions provided with the kits.

#### 2.11.23 Energy metabolism biomarkers

Serum levels of Sirtuin 1 (Sirt1), Forkhead box O3 (FOXO3), and Perilipin 2 (PLIN2) will be determined by ELISA using kits from Jiangsu Enzyme Exemption Industry Co., Ltd. (MM-13492H1, MM-0310H1, and MM-60717H1, respectively). Concurrently, serum cortisol levels will be measured using a kit from Nanjing SenBeijia Biological Technology Co., Ltd. (SBJ-H0998-96T). The specific experimental procedures will be carried out in strict accordance with the instructions provided with the kits.

### 2.12 Statistical analysis

All statistical analyses will be performed using R software (version 4.3.1 or later; R Foundation for Statistical Computing, Vienna, Austria). For the description of baseline data, normally distributed continuous variables will be presented as mean±standard deviation (SD), while non-normally distributed variables will be reported as median [interquartile range (IQR)]; categorical variables will be expressed as frequencies and percentages (n, %). For the comparison of baseline data between groups, independent samples t-tests will be used for normally distributed continuous variables, the Mann-Whitney U test will be used for non-normally distributed data, and the chi-square (χ²) test or Fisher’s exact test will be used for categorical data.

Efficacy analyses will be conducted according to the intention-to-treat (ITT) principle and will include all randomized participants. Continuous outcomes, including NMD and GWE, will be analyzed using linear mixed-effects models (LMMs). To rigorously control for potential confounding, background medication use will be prespecified and included as fixed-effect covariates in the models. Medications will be categorized by therapeutic class, with binary indicators (yes/no) created for: (1) glucose-lowering agents (with separate indicators for GLP-1 receptor agonists and SGLT2 inhibitors), (2) antihypertensive medications, and (3) statins. Each LMM will incorporate group, time, and the group×time  interaction as fixed effects, with a random intercept for each participant to account for repeated measurements.

Sensitivity analyses will be performed using multiple imputation to assess the robustness of the findings under different assumptions regarding missing data. In addition, a per-protocol (PP) analysis will be conducted as a secondary sensitivity analysis, including only participants with an attendance rate of at least 80%. Secondary outcomes will be considered exploratory and hypothesis-generating; therefore, no adjustment for multiple comparisons will be applied. A two–tailed *P*–value < 0.05 will be considered statistically significant.

## 3 Discussion

Energy metabolism is a complex and highly regulated dynamic process. Patients with T2DM are characterized by metabolic inflexibility, which manifests as widespread dysregulation of glucose, lipid, and protein metabolism. AE is a well-established non-pharmacological intervention recognized for its clinical benefits in ameliorating these metabolic abnormalities (24). Given the dynamic nature of energy metabolism, this study will utilize indirect calorimetry to systematically assess systemic energy metabolism in patients with T2DM, including key parameters such as resting energy expenditure, substrate oxidation rates, and metabolic flexibility. Furthermore, considering that humoral regulation plays a central role in maintaining metabolic homeostasis and is frequently disrupted in T2DM, this study will also quantify the circulating levels of cortisol, as well as Sirt1 and FOXO3, which are crucial regulators of cellular energy metabolism.

In addition to metabolic dysregulation, systemic inflammation and oxidative stress are key drivers of the pathological remodeling and cardiovascular dysfunction observed in patients with T2DM. Acknowledging the central role of these processes, this study will comprehensively assess the impact of AE on systemic inflammation and oxidative stress by quantifying a panel of circulating biomarkers, including oxidative stress markers (ROS, SOD, MDA, Nrf2, HO-1, GPX4, NQO1) and inflammatory biomarkers (IL-18, IL-1β, IL-6, TNF-α, CRP, NLRP3). Furthermore, while AE has been shown to ameliorate myocardial remodeling and improve diastolic dysfunction in patients with T2DM, evidence regarding its effects on systolic function remains inconsistent (25, 26). This inconsistency may be partly attributable to the limited sensitivity of conventional systolic indices, such as left ventricular ejection fraction (LVEF), for detecting the subclinical contractile dysfunction that often characterizes early-stage T2DM cardiomyopathy (27). Global longitudinal strain (GLS) has emerged as a more sensitive metric for detecting subclinical myocardial dysfunction, making it a more appropriate endpoint for evaluating systolic responses to AE. This approach is further supported by our previous preclinical work, which demonstrated that AE improves radial strain in diabetic rats (28). Accordingly, this study will utilize GLS to investigate the effects of AE on systolic function in patients with T2DM. Finally, it is critical to recognize that beyond structural and functional impairments, T2DM also induces myocardial metabolic inflexibility. While preclinical studies have shown that AE can ameliorate this metabolic rigidity, clinical evidence in humans remains scarce. Therefore, this study will utilize the novel index of global work efficiency (GWE) to assess whether AE can improve myocardial energetic efficiency in patients with T2DM (29).

Beyond its profound impact on the heart, T2DM also induces pathological vascular remodeling, characterized by arterial wall thickening and increased arterial stiffness, which elevates the risk of atherosclerosis (30). Vascular tone is co-regulated by endothelium-dependent and endothelium-independent (smooth muscle–mediated) mechanisms. Although some evidence suggests that AE can partially ameliorate T2DM-induced endothelial dysfunction, its effects on vascular smooth muscle function remain largely unknown (31). Accordingly, this study will assess NMD to investigate the effects of AE on the endothelium-independent pathway.

The cardiovascular system is governed by complex neurohumoral regulatory mechanisms, and imbalances in these pathways are closely linked to the cardiovascular complications of T2DM. Persistent, low-grade inflammation and oxidative stress can elevate circulating levels of epinephrine and norepinephrine in patients with T2DM. To explore whether AE exerts its cardiovascular protective effects via the modulation of these neurohumoral pathways, this study will measure circulating epinephrine and norepinephrine levels before and after the intervention. Given that catecholamine dysregulation is a key driver of hemodynamic instability, while AE is a well-established intervention for improving blood pressure in T2DM, blood pressure is a dynamic variable with a distinct circadian rhythm. Single, static office blood pressure readings cannot adequately capture overall blood pressure homeostasis. In contrast, 24-hour ambulatory blood pressure monitoring (ABPM) provides a comprehensive, dynamic blood pressure profile, detailing diurnal and nocturnal levels, circadian patterns, and blood pressure variability, allowing for an integrated analysis of neurohumoral and hemodynamic adaptations.

Peak oxygen uptake (VO_2peak_) represents the maximal capacity of this entire integrated process and is one of the most powerful predictors of cardiovascular events and all-cause mortality. Previous studies have consistently shown that even in patients with T2DM who are free of overt cardiovascular disease, VO_2peak_ is reduced by approximately 20–30%, indicating that integrative physiological dysfunction is an early feature of the disease (32). Existing research regarding the effects of AE on VO_2peak_ in T2DM has largely focused on observing improvements in single parameters. While confirming the presence of global functional impairment, this approach has been insufficient to pinpoint the predominant mechanisms responsible for the exercise-induced improvements. Therefore, by concurrently measuring VO_2peak_ and its underlying determinants, this study aims to deconstruct the systemic physiological response into its constituent multi-system components. This will allow us to determine whether improvements in VO_2peak_ following AE are primarily driven by enhanced cardiac function, augmented skeletal muscle oxygen utilization, restored vascular perfusion, or improved autonomic regulation.

Physical inactivity is a common behavioral characteristic of patients with T2DM. This study will therefore utilize accelerometry to objectively monitor 24-hour movement behavior and evaluate the impact of the AE intervention on daily activity patterns of patients with T2DM. Fatigue is a key contributor to this inactivity, but is often overlooked in the design of clinical exercise prescriptions, representing a significant barrier to intervention adherence. The pathogenesis of fatigue in T2DM is multifactorial, involving both central and peripheral mechanisms. Central fatigue is linked to neurotransmitter imbalances, manifesting as reduced central drive. This study will therefore assess the central fatigue state by quantifying circulating levels of key neurotransmitters, including serotonin (5-HT), dopamine (DA), and γ-aminobutyric acid (GABA) (33). Peripheral fatigue, in contrast, is primarily associated with skeletal muscle dysfunction (34). To assess the state of skeletal muscle and peripheral fatigue, this study will measure serum levels of creatine kinase (CK) and myoglobin (Myo). Given the paucity of robust clinical evidence in this area, this dual-pathway assessment aims to systematically elucidate the specific physiological mechanisms by which AE alleviates fatigue in patients with T2DM.

T2DM is a complex metabolic disorder that precipitates metabolic dysregulation and cardiovascular dysfunction at both the cellular and organ levels (35), which in turn progresses to systemic impairments in metabolism and cardiovascular health (4, 36). While AE has been shown to improve the function of individual organs and specific metabolic pathways (37), it remains unclear whether these discrete benefits translate into an improved state of integrated, whole-body physiological function. Therefore, the present study is designed to bridge the gap between organ-level mechanisms and systemic outcomes, integrating the potential mechanisms at the cellular and organ levels to explain AE-mediated functional improvements from a whole-body perspective, aiming to elucidate how AE promotes metabolic and cardiovascular health from an integrative physiological perspective. This approach may provide novel insights for the optimization of clinical exercise management in patients with T2DM.

## 4 Limitations

Several potential limitations of this trial warrant acknowledgment. First, a central, unresolved issue in exercise medicine is the optimal dose-response relationship for health benefits in T2DM. Due to practical constraints, this study will investigate only a single exercise modality and intensity, which precludes the identification of the optimal exercise prescription for improving integrated cardiovascular and metabolic health in this population. Second, although this study includes a comprehensive panel of cardiovascular and metabolic indices, the exploration of in-depth molecular mechanisms is beyond the scope of this clinical trial design. Third, as a single-center study involving community-dwelling Chinese adults, the generalizability of the findings to other ethnic groups or distinct healthcare systems may be limited. Fourth, the current study design lacks a long-term follow-up period beyond the 12-week intervention, which prevents the assessment of the durability of the intervention’s effects. Finally, given the large number of secondary and exploratory biomarkers assessed, we acknowledge the inherent risk of Type I errors; consequently, findings related to these exploratory outcomes should be interpreted as hypothesis-generating rather than definitive. Despite these limitations, this trial is expected to provide valuable insights into the mechanisms by which AE promotes integrated cardiovascular and metabolic function in patients with T2DM.
